# Face Validity of the Single Work Ability Item: Comparison with Objectively Measured Heart Rate Reserve over Several Days

**DOI:** 10.3390/ijerph110505333

**Published:** 2014-05-16

**Authors:** Nidhi Gupta, Bjørn Søvsø Jensen, Karen Søgaard, Isabella Gomes Carneiro, Caroline Stordal Christiansen, Christiana Hanisch, Andreas Holtermann

**Affiliations:** 1 National Research Centre for the Working Environment, 2100 Copenhagen, Denmark; E-Mails: bjorn@multitesta.dk (B.S.J.); igc@nrcwe.dk (I.G.C.); carolinestordal@gmail.com (C.S.C.); aho@nrcwe.dk (A.H.); 2 Institute of Sports Science and Clinical Biomechanics, University of Southern Denmark, 5230, Odense M, Denmark; E-Mail: ksogaard@health.sdu.dk; 3 Federal Institute for Occupational Safety and Health (BAuA) Nöldnerstr, 40-42, D-10317, Berlin, Germany; E-Mail: hanisch.christiana@baua.bund.de

**Keywords:** work ability, face validity, relative aerobic workload, blue-collar workers, actiheart, objective measures

## Abstract

*Purpose*: The purpose of this study was to investigate the face validity of the self-reported single item work ability with objectively measured heart rate reserve (%HRR) among blue-collar workers. *Methods*: We utilized data from 127 blue-collar workers (Female = 53; Male = 74) aged 18–65 years from the cross-sectional “New method for Objective Measurements of physical Activity in Daily living (NOMAD)” study. The workers reported their single item work ability and completed an aerobic capacity cycling test and objective measurements of heart rate reserve monitored with Actiheart for 3–4 days with a total of 5,810 h, including 2,640 working hours. *Results*: A significant moderate correlation between work ability and %HRR was observed among males (*R* = −0.33, *P* = 0.005), but not among females (*R* = 0.11, *P* = 0.431). In a gender-stratified multi-adjusted logistic regression analysis, males with high %HRR were more likely to report a reduced work ability compared to males with low %HRR [OR = 4.75, 95% confidence interval (95% CI) = 1.31 to 17.25]. However, this association was not found among females (OR = 0.26, 95% CI 0.03 to 2.16), and a significant interaction between work ability, %HRR and gender was observed (*P* = 0.03). *Conclusions*: The observed association between work ability and objectively measured %HRR over several days among male blue-collar workers supports the face validity of the single work ability item. It is a useful and valid measure of the relation between physical work demands and resources among male blue-collar workers. The contrasting association among females needs to be further investigated.

## 1. Introduction

In the working population, unskilled and semi-skilled workers (blue-collar workers) generally have an elevated risk of premature drop-out from the labor market [1]. This is considered to be a result of excessive work demands in relation to the resources among these workers [2]. A valid and simple tool for workload monitoring, screening and modifications based on the relation between the work demands and resources of the worker has therefore been extensively requested.

A frequently used tool in research and practice for assessing the balance between work demands and resources of the worker is the concept of work ability [3]. When the workers resources do not exceed the demands at work with a certain safety margin, this may be expressed as decreased work ability [4]. Accordingly, reduced work ability is related to self-reported high physical work demands and low physical resources [5], long-term sickness absence, and early retirement from the laboring market [6]. Therefore, the concept of work ability is a well-recognized tool for work environment related research and practice.

One of the earliest developed and most commonly used instruments to measure work ability is the Work Ability Index (WAI), which originally includes a series of questions dealing with seven dimensions of work characteristics and health [6,7]. However, because of the relatively complex design of the WAI and related practical issues, a single-item measure of work ability has generally been preferred for larger surveys [7,8]. This single item measure of work ability has also been shown to predict short and long-term sickness absence [7,9] and correspond well to a complete work ability index based on several questions [10].

Few studies have analyzed the relation of work ability with work demands and worker’s resources [5,11,12]. However, it remains unknown how well the single item work ability instrument reflects the balance between work demands and resources of the worker (*i.e.*, the face validity) [13]. Because the work ability conceptually corresponds to the balance between the work demands and resources of the worker [3], a good face validity of the work ability instrument would require a good correspondence to other measures reflecting this balance between work demands and resources.

However, previous studies have mainly used self-reported measures of work demands, which generally have limited reproducibility and validity [14]. Also, workers with lowered work ability are likely to overestimate their self-reported work demands, compared to those with higher work ability [6]. Moreover, the perceived work ability may be subjected to self-reporting bias from factors like socioeconomic status, which may explain some of the strong predictive value for work on health-related issues [15]. We therefore find it important to investigate how well the single-item work ability corresponds with an objective measure reflecting the balance between work demands and resources among a population of workers with similar socioeconomic status such as blue-collar workers.

A physiologically similar concept to work ability is the heart rate reserve (% HRR) which is a well-recognized and validated objective measure of the balance between the work demands and resources of the worker [16]. %HRR is expressed as the percentage of the range between resting and maximal heart rate [17]. %HRR method is preferred over others methods to express worker’s workload relative to his resources because it is based on measured heart rate during several work hours which reflects the physical demands as well as psychosocial stressors [18]. Moreover, because it utilizes both the maximal heart rate which depends on age [19] and resting heart rate which depends on physical resources (*i.e.*, physical fitness) [20], %HRR is applicable among workers with varying age and physical resources [21]. Thus, %HRR is well documented to provide a measure of the physiological cardiovascular strain on the body depending on the work demands and the resources of the worker [22].

Therefore, the primary aim of this study was to investigate the face validity of the self-reported single work ability item by investigating its association with the objectively measured %HRR over several days among blue-collar workers from the New method for Objective Measurements of physical Activity in Daily living (NOMAD) study. We hypothesized that the single item perceived work ability has a relatively good face validity compared to the physiological similar concept of %HRR among blue-collar workers.

## 2. Methods and Analysis

### 2.1. Study Design and Population

A cross-sectional study was conducted on blue-collar workers recruited from seven workplaces in Denmark (construction workers, cleaners, road maintenance workers, street cleaners/garbage disposers, manufacturing workers, truck drivers and workers in the health service sector). The inclusion criterion at workplace level was the possibility for the workers to participate in the study activities during paid working-time. Inclusion criteria for participating in the study were primary work (main occupation) ≥20 h per week and being between 18 and 65 years of age. Exclusion criteria for taking part in the study were declining to sign the informed consent, pregnancy or sickness on the day of testing. Furthermore, allergy to band aid caused exclusion from the objective field measurements, whereas hypertension (≥160/≥100 mmHg), angina pectoris, medical treatment of heart and lungs, and/or trauma/serious pain in involved body parts led to exclusion from specific aerobic capacity test. The study was approved by the Ethics Committee for the capital region in Denmark (journal number H-2-2011-047) and conducted in accordance with the Helsinki declaration.

### 2.2. Procedure

Data recording was conducted over four days with research staff visiting the workers at the workplace on day 1 and 4. On day 1, workers interested in participating in the study were invited for: (a) anthropometric and aerobic capacity measurements; (b) objective heart rate measurements; and (c) completing a computer-based questionnaire. On day 4, the workers returned back the objective measurement devices.

#### 2.2.1. Anthropometric and Aerobic Capacity Measurements

The anthropometric measurements of the workers including height (model 123, Seca, Birmingham, UK) and body weight (model BC418 MA, Tanita Corporation, Tokyo, Japan) were taken and their body mass index (BMI, kg/m^2^) was calculated. The aerobic capacity of each worker was estimated using a one point Åstrand sub-maximal test on a bicycle ergometer (model 874E, Monark, Stockholm, Sweden) [23]. The worker was instructed to maintain a cadence at 60 rounds per minute throughout the test at a resistance chosen based on age, self-reported physical fitness and cycling habits of the worker. After two minutes, the workload was increased to elicit a steady—state heart rate of ≥60% of maximal heart rate [19]. Heart rate was recorded continuously and the age and gender corrected aerobic capacity (mL O_2_/min/kg) was estimated by the Åstrand nomogram, based on steady state HR and corresponding workload during the ergometer bicycle test. This test is generally shown to be a valid measure of aerobic capacity [24], feasible to be performed among blue-collar workers at the work-site [25], and strongly influence the association between physical work demands and cardiovascular and all-cause mortality [26].

#### 2.2.2. Questionnaire

Self-reported information about single item work ability was obtained by the following question modified from the original WAI: “*How many points will you give to your current work ability*”. The work ability was measured in points ranging from 0 (not capable of working) to 9 (best work ability) [25,27]. To facilitate the discussion, the work ability scale in present study was stratified into the categories as “good work ability” (≥8) and “reduced work ability” (<8) [28,29]. Smoking behavior was analyzed using a question: “*Do you smoke?*” with four response categories summarized into two groups; yes (yes daily; yes sometimes) and no (used to smoke, not anymore; and I have never smoked); for analysis. Seniority in total months was determined with the question: “*For how long have you had the kind of occupation as you have now?*” The influence at work (decision authority) of the workers was determined by the four items scale from Copenhagen Psychosocial Questionnaire [30]. A sample item: “*Do you have a large degree of influence concerning your work?*” and Cronbach’s Alpha of the four items: 0.77. The responses were scored on a Likert scale with answer categories ranging from 0 (never) to 5 (always). An influence at work scale was composed as the mean of all four items. For analysis, this scale was recorded to 0–100 scale with 100 representing the highest degree of influence at work. The age, seniority, BMI, and influence at work were treated as continuous variables and smoking, work ability and %HRR as categorical variables in the analysis.

#### 2.2.3. Heart Rate Reserve

A compact device, Actiheart (CamNtech Ltd., Cambridge, UK; sensitivity: 250 µV), was utilised to determine heart rate to estimate %HRR. The Actiheart worn on the chest consisted of two electrodes connected by a short lead which clip onto two standard electrocardiography (ECG) pads. After a light preparation of the skin (cleaning the skin with 73% ethanol spirits), the ECG electrodes (Ambu^©^, Blue sensor VL-00-S/25) were placed on the chest at the standardized position explain by Kristiansen *et al.* [31]. The start time was specified in agreement with the workers, followed by approximately 3–4 consecutive days of recording, ideally a period covering at least two working days. The workers were instructed to remove the heart rate measurement device if it caused itching or discomfort. During the measurements, all workers were instructed to fill out a diary, in which they daily noted the time of getting up in the morning, going to bed at night, start of work, and end of work.

The workers who did not have at least one work-day of valid objective heart rate measurements (defined as ≥ 4 h/day and ≥ a total of 7 h during all recorded working periods) were excluded from the main analysis. The reason behind choosing these criteria is: (a) to ensure the inclusion of the workers who were at least working 20 h/week with an average of 4 hours/day and (b) to ensure the inclusion of at least one work-day per person. The appropriateness of using one valid day of measurement has been discussed elsewhere [32].

Using band-pass filtration device (10–35 Hz), the analog signals of the Actiheart recorder were filtered, sampled with a frequency of 128 Hz, and processed by a real time QRS-detection algorithm. A custom made software, Acti4, (National Research Centre for the Working Environment, Copenhagen, Denmark, and Federal Institute for Occupational Safety and Health, Berlin, Germany) was utilized to analyze Actiheart’s inter-beat objective measures of the heart rate during physical activity at work [31]. Since the R wave to R wave (RR) intervals stored in the Actiheart recorder were not filtered beforehand, a thorough filtering was done with the following criteria: the inter-beat intervals were considered erroneous and rejected from the analysis if these intervals were too short or too long. Thereafter, if the beat error is more than 50% of the beats within 10 sec time intervals, all beats in that interval were rejected and similar procedure was repeated for 50 sec time intervals. Subsequently, the mean heart rate was calculated for each work interval, if the beat error is lower than 50% for that particular interval. The heart rate for all work intervals was calculated by interpolating the heart rate data with the frequency of 4 Hz.

#### 2.2.4. Data Processing and Calculation of Heart Rate Reserve

In this study, heart rate reserve (%HRR) at work was defined as [17,33]:

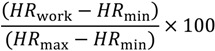

*HR*_min_ was defined as the minimum value of a running average of ten heart beats throughout all heart rate values in the measurement period. Maximal heart rate (*HR*_max_) was estimated utilising the equation (*HR*_max_ = 208 − 0.7 × age) by Tanaka, Monahan and Seals [19]. Generally, a linear relation exists between the heart rate and the oxygen utilized during a work task, regardless of age and sex [34]. Therefore, at group level, %HRR is closely related to relative aerobic workload [35] which measures relative load in terms of the absolute oxygen uptake during work as a percentage of maximal oxygen uptake [17]. A recommended threshold for the maximal average relative aerobic workload during 8-hour workday is 33% [22,36,37]. Therefore we applied this threshold of 33% to stratify %HRR into the categories “*low*” (≤33% HRR) and “*high*” (>33% HRR). Working below this threshold is considered to generally ensure a healthy balance between the work demands and resources of the worker, while exceeding this threshold would lead to excessive exhaustion and fatigue [22,38]. Because of varying working hours between the workers, a sensitivity analysis was performed using tailored cut-points of %HRR depending on number of work hours [38]. Specifically, the workload was defined as *high* if the workers had: (a) 4 working hours per day and >45% HRR; (b) 8 working hours per day and >33% HRR; (c) 10 working hours per day and >30.5% HRR; and (d) more than 10 working hours per day and >28%HRR [38].

### 2.3. Statistical Analyses

To investigate the face validity of single item self-reported work ability, we analyzed our data using Spearman rank correlation between categorical scores of work ability and %HRR. Moreover, the association between work ability as the dependent variable; [categorized into *reduced* (<8) and *good* work ability (≥8)] and %HRR (“*low* HRR” ≤ 33% and “*high* HRR” > 33%) as the independent variable was investigated with a binary logistic regression model. The model was step-wise adjusted for the following covariates; step 1: age and gender; step 2: step 1 + body mass index (BMI), smoking, seniority; step 3: step 2 + influence at work; and step 4: step 3 + aerobic capacity. Moreover, the previously described sensitivity analysis with tailored cut-points of %HRR depending on number of working hours was performed using the same statistical model with step-wise entry of covariates. The odds-ratios (OR) were estimated indicating the odds of reporting a *reduced* work ability (<8) compared to *good* work ability (≥8). As the effect modification due to gender on the association of %HRR and work ability was significant [%HRR × gender, OR = 7.327 (95%CI = 1.19–45.15), *P* = 0.03], separate logistic regressions were performed for each gender. Additionally, to test the robustness of the results obtained from OR, a separate sensitivity analysis was performed using log binomial regression to calculate crude relative risk (RR, with adjustment of only age, step 1). Statistical Package for the Social Sciences (IBM Corporation SPSS statistics, version 21.0, Armonk, NY, USA) was utilized to perform all statistical operations. The level of significance was set at *P* < 0.05.

## 3. Results

**Figure 1:**
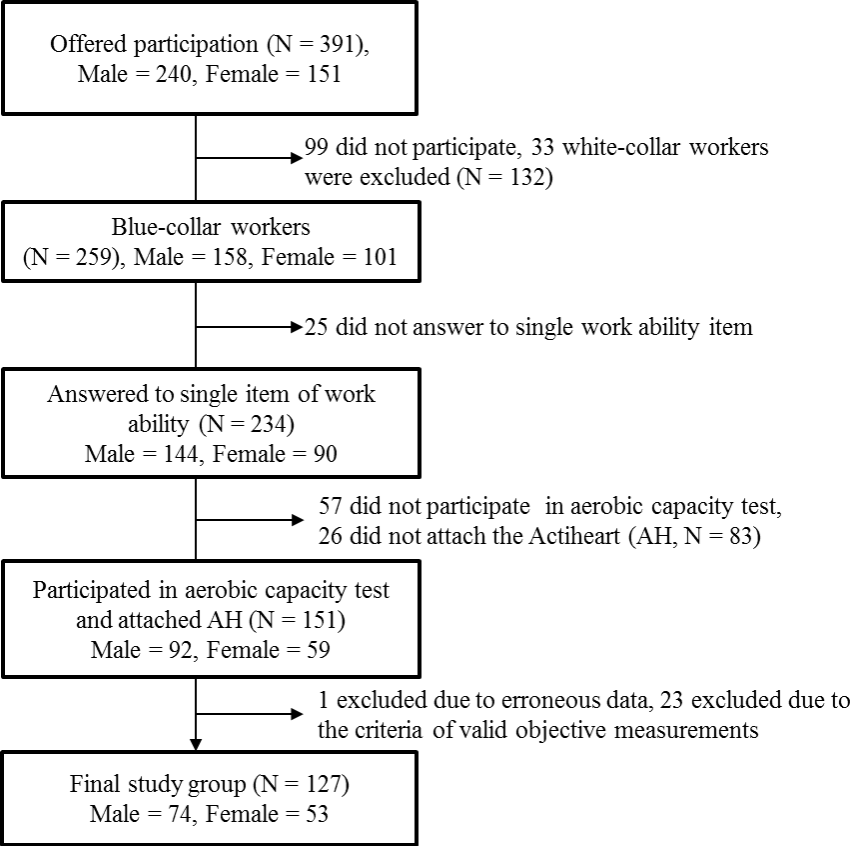
Flow diagram of the study group.

Of 391 workers, 259 blue-collar workers volunteered to participate in the study. Of them, 151 workers answered to at least the single work ability item, participated in objective heart rate measurements, and the aerobic capacity test. Of them, only one worker was excluded due to erroneous heart rate data. Ten workers were excluded due to their normal reported working hours being <4 hours/day and 13 workers were excluded due to insufficient (<7 h) total working hours on all measured days. Therefore, 127 workers were included in the main analysis (Figure 1). Their descriptive variables have been summarized in Table 1 and Table 2. Males were predominantly working as manufacturing laborers (49%) or mining and construction laborers (30%). However, female workers were primarily industrial assemblers (38%).

The average recorded heart rate during working hours was 89 bpm (SD = 10) with maximal heart rate of 179 bpm (SD = 7 bpm) among males and 88 bpm (SD = 10) and 176 bpm (SD =6 bpm), respectively, among female workers.

Table 1 shows the characteristics of the male and female workers stratified on their work ability. Males with reduced and good work ability were similar with respect to most characteristics except for their %HRR, age and influence at work. The male workers with reduced work ability were younger, exposed to high %HRR and reported low influence at work, compared to those with good work ability. Similarly, the female workers with reduced work ability had low influence at work compared to those with good work ability. Moreover, these female workers with reduced work ability had a slightly lower %HRR compared to those with good work ability.

**Table 1:** Demographic characteristics of the male and female workers based on the single item self-reported work ability item.

Variables	Male		Female	
	Reduced Work ability (*n* = 24)	Good Work ability (*n* = 50)	Reduced Work ability (*n* = 11)	Good Work ability (*n* = 42)
Age in years (M, SD)	37.6 (9.5)	43.0 (9.6)	45.6 (7.4)	45.7 (8.2)
Seniority in months (M, SD)	112.0 (114.9)	145.3 (127.7)	150.1 (105.6)	146.7 (134.1)
BMI in kg/m^2^ (M, SD)	25.4 (3.5)	25.7 (3.2)	26.0 (5.2)	25.7 (6.1)
Current smokers (%)	45.5	45.8	40.0	53.8
HRR in % (M, SD)	35.8 (6.8)	31.0 (6.7)	28.8 (6.1)	31.6 (6.6)
Aerobic capacity in mL/kg/min (M, SD)	34 (10)	34 (8)	29.1 (7)	29 (7)
Influence at work (M, SD)	33.9 (19.7)	43.4 (19.9)	33.0 (33.2)	43.8 (24.1)

Notes: Reduced work ability <8 and good work ability ≥8 on a scale from 0 (not capable of working) to 9 (best work ability); BMI = body mass index; M = mean; *n* = number of workers; SD = standard deviation; According to the recommendation of the STROBE statement [39], no significance test to discriminate between reduced and good work ability groups was performed.

Table 2 shows the characteristics of the workers stratified on low and high % HRR. The male workers with high and low %HRR were similar on most characteristics except for aerobic capacity which was lower among males with high % HRR. However, female workers with high and low %HRR differed in seniority, BMI, percentage of smokers, work ability and influence at work. Specifically, the female workers with high %HRR had a lower seniority, higher BMI and percentage of smokers, slightly higher work ability, and lower influence at work, compared to those with low % HRR.

**Table 2:** Demographic characteristics of the male and female workers stratified on %HRR.

Variables	Male		Female	
	Low %HRR (*n* = 39)	High %HRR (*n* = 35)	Low %HRR (*n* = 33)	High %HRR (*n* = 20)
Age in years (M, SD)	42.3 (9.1)	40.1 (10.7)	45.5 (8.4)	46.0 (7.6)
Seniority in months (M, SD)	151.9 (132.8)	114.8 (111.6)	182.1 (139.7)	75.8 (50.4)
BMI in kg/m^2^ (M, SD)	25.4 (3.0)	25.8 (3.5)	24.4 (5.1)	28.0 (6.3)
Current smokers (%)	47.2	44.1	40.6	70.6
Work ability (M, SD)	8.0 (0.8)	7.7(1.1)	7.8 (1.1)	8.3 (0.7)
Aerobic capacity in mL/kg/min (M, SD)	37 (9)	32 (7)	30 (6)	27 (8)
Influence at work (M, SD)	41.0 ( 20.5)	39.5 (20.1)	45.6 (27.9)	34.7 (22.3)

Note: Low HRR ≤ 33%, high HRR > 33%; BMI = body mass index; HRR = heart rate reserve; M= mean; *n* = number of workers; SD = standard deviation; According to the recommendation of the STROBE statement [39], no significance test to discriminate between low and high HRR groups was performed.

There was a significant moderate correlation between work ability and %HRR among males (R = −0.33, *P* = 0.005) but not among females (R = 0.11, *P* = 0.431).

**Figure 2:**
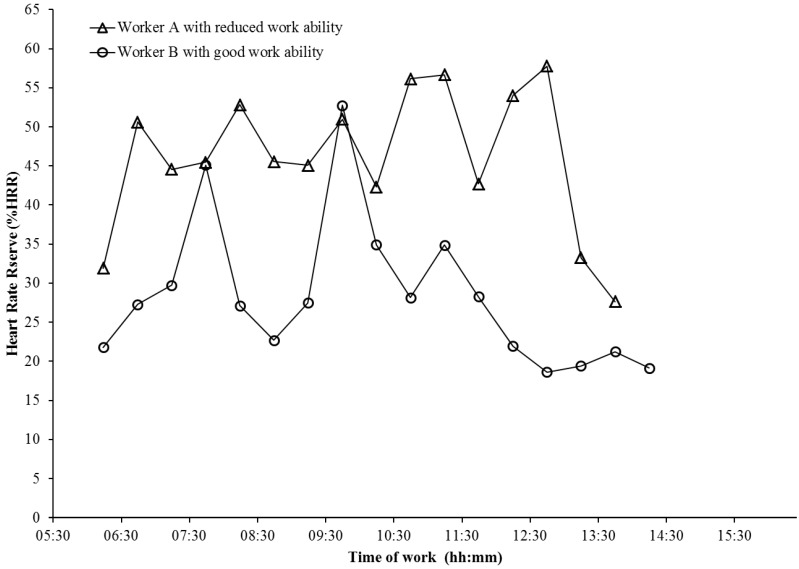
Exemplary visual representation of the heart rate reserve (%HRR) for two workers from the same workplace with similar work hours, but different work ability (male worker A with reduced work ability and male worker B with good work ability).

Table 3 shows the gender stratified results of the association between %HRR and work ability. The male workers with high %HRR had a significantly higher probability of reporting *reduced* work ability compared to those with low %HRR. However, a non-significant association in the opposite direction was found among female workers. Adjustment for various confounders such as age, BMI, seniority, smoking, influence at work and aerobic capacity did not materially influence the estimates for both genders. A similar pattern is exemplified in Figure 2 of two male workers with similar number of work hours and working at the same workplace, but with different work ability. As seen in the figure, the %HRR of worker A with reduced work ability was constantly higher compared to worker B with good work ability.

The results of crude RR (adjusted for age) obtained with log binomial regression were somewhat weaker than the OR among both genders. The crude relative risk for reduced work ability was 2.41 (CI = 1.12 to 5.20) among males with high %HRR and 0.62 (CI 0.19 to 2.07) among females with high %HRR.

The sensitivity analyses with tailored cut-points of %HRR depending on number of work hours adjusted for age, BMI, seniority, smoking, influence at work and aerobic capacity showed very similar estimates of the association between work ability and %HRR as in the primary analysis with a fixed cut-point at 33% of HRR among both genders (males: OR = 4.83, 95%CI 1.03 to 22.71, females: OR = 0.11, 95%CI 0.01 to 0.96).

**Table 3:** Logistic regression model estimating the association between heart rate reserve measured for 3–4 days and reduced work ability among males (*n* = 75) and females (*n* = 53).

Steps	%HRR	Male	Female
		OR (95%CI)	OR (95%CI)
Step 1 ^a^	Low	1 ^e^	1 ^e^
	High	4.11 * (1.39 to 12.11)	0.55 (0.13 to 2.38)
Step 2 ^b^	Low	1 ^e^	1 ^e^
	High	4.63 ** (1.45 to 14.75)	0.32 (0.04 to 2.30)
Step 3 ^c^	Low	1 ^e^	1 ^e^
	High	4.98 ** (1.49 to 16.69)	0.30 (0.04 to 2.30)
Step 4 ^d^	Low	1 ^e^	1 ^e^
	High	4.75 * (1.31 to 17.25)	0.26 (0.03 to 2.16)

Notes: * *P* ≤ 0.05; ** *P* ≤ 0.01; ^a^ Adjusted for age (step 1); ^b^ Adjusted for step 1 and seniority, smoking and BMI (step 2); ^c^ Adjusted for step 2 and influence at work (step 3); ^d^ Adjusted for step 3 and aerobic capacity (step 4); ^e^ Reference; HRR = heart rate reserve, high HRR > 33%, low HRR ≤ 33%, reduced work ability <8 on a scale from 0 (not capable of working) to 9 (best work ability).

## 4. Discussion

The main finding in this study was the relatively strong (according to the qualitative descriptors of effect size for the odds ratio) [40] negative association between %HRR and work ability among male workers. Also we observed an expected negative correlation between work ability and %HRR, *i.e.*, the male workers with high %HRR during working hours were more likely to report reduced work ability. These results confirm that the work ability and %HRR reflects a common construct, considered to be the balance between the work demands and resources of the worker [5,16]. To the best of our knowledge, this is the first study to determine the face validity of the self-reported single work ability item against another objective method reflecting this balance between the work demands and resources of the worker. The correlation between work ability and objectively measured %HRR was 0.33 indicating a moderate correspondence between the work ability and the %HRR. The reasoning for the correlation not being higher, may be due to the multi-dimensional nature of work ability [6], not being completely covered by the measured %HRR in this study.

Among females, a tendency towards a positive association between work ability and %HRR was found. In other words, females with high %HRR had a higher tendency of reporting good work ability compared with females with low %HRR. These findings indicate a different direction of the association between work ability and %HRR between the genders. An explanation for this result could be that the distribution of work tasks requiring high physical workload may be different among male and female workers. For example, it may be a higher degree of tailoring of the work tasks (*i.e.*, fitting the work task to the work ability of the worker) in female-dominated occupations compared with male-dominated occupations. The frequency distributions may support this explanation, showing that among all workers with reduced work ability, a relatively small proportion (27%) of female workers was exposed to high %HRR compared to male workers (71%). However, if this explanation holds needs to be investigated in future studies.

The OR tends to overestimate the magnitude of RR if the prevalence of the outcome is high [41]. In this study, the prevalence of the outcome reduced work ability was relatively high (27.6%). Therefore, we also performed a sensitivity analysis to estimate the RR instead of OR as a measure of association between work ability and %HRR. As expected, we obtained a slightly reduced RR compared with the OR for both genders. For example for males, the RR was 2.41 compared to OR of 4.11.

### 4.1. Strength and Limitations

The primary strength of this study was the use of the objective measurement of %HRR to determine face validity of work ability. The heart rate measurement device used in this study is water resistant and fitted firmly to the skin during the measurement period. Thus, no special care for the equipment is required and the workers could perform their work without any interruption and uneasiness. These objective measurement devices such as Actiheart have been utilised previously on workers with physically demanding jobs [31]. Another strength is the data consisting of %HRR measures from nearly 5,810 h, which included as many as 2,640 working hours.

However, some limitations should be considered when interpreting the results in this study. The workers included in the statistical analysis of this study represent a relatively small percentage of the workers who offered participation (32.5%). Furthermore, we utilized prediction equation to estimate maximal heart rate (HR_max_) in this study which may add some variability to the results. However, Tanaka, Monahan and Seals [19] indicated that a large proportion of the variability in estimating the HR_max_ is explained by the variables in the prediction equation. Robergs and Landwehr [42] also considered Tanaka and Collegues’ [19] method of estimating HR_max_ as the most accurate prediction method. Another limitation could be that the estimation of the thresholds of low and high %HRR based on relative aerobic workload may not be precise due to the relation between HR and oxygen uptake not being linear at very low (slightly above resting HR) and very high intensity of work tasks [43]. However, in this study, the average HR during working periods among all workers was 89 bpm with a SD of 10 bpm, indicating that the main fraction of the measured HR is within the valid range of HR. Furthermore, we utilized broad categories of low (≤33%) and high HRR (>33%) which make the finding less sensitive for some imprecision of the estimated %HRR. Finally, we do not know if the observed associations between %HRR and the work ability measured with the single item is also applicable for the entire work ability scale.

### 4.2. Methodological Considerations

We utilized a modified version of single item of perceived work ability where we asked workers to rate their current work ability on a scale of 0 (not capable of working) to 9 (best work ability). Work ability is a very stable variable unlike other variables such as muscle pain which highly fluctuates with short time [44]. Thus, a specific time point is not included in the work ability question. Based on our personal experiences, we observed that workers generally have difficulties in conceptualizing the word “lifetime best” from original scale. Thus, we used a modified version of single item work ability which has also been used previously [25,45]. Additionally, the validity of scale of work ability ranging from 0–9 has been shown to have a good agreement with original work ability scale from 0–10 [25].

The used cut points of reduced (<8) and good (≥8) work ability are based on previous studies [28,29]. By using these cut points, the prevalence of reduced work ability in this study is 27.6%, which seems realistic in a study population of blue-collar workers with rather high seniority and age. If we shift our cut point of “reduced work ability” one point down at <7, the prevalence of reduced work ability would be around only 9% which is even lower than the reported prevalence of reduced work ability among a representative population of the workers (combining white and blue-collar workers) [7,28,46].

Very few previous studies have assessed the validity of the single item work ability [47]. Some of these studies have demonstrated that the single item work ability is associated with employability, work environment, and work ability index [10,48,49]. However, these variables were measured with self-report which generally have a number of limitations [14]. %HRR is known to be a precise objective measure of the workload relative to available resources of a worker, particularly in dynamic work tasks and with lesser precision in static work tasks [50]. However, the work tasks of the workers in the present study being mainly characterized by dynamic work, the extensive measured period during working hours, (21 h per person on average), and the broad cut points utilized to categorize %HRR of the workers into high and low HRR (33% HRR) are considered to minimize the potential impact of imprecision of the measured %HRR during static work in this study.

Several studies have suggested a cut point of 33% relative aerobic workload as acceptable workload for a general 8 h physical work day [22,36,37]. However, it is recommended that this cut point of safe relative workload is adjusted to the number of working hours [35]. The number of working hours of all workers in this study varies from 4–16 h. Thus, we performed a separate analysis based on tailored cut points of %HRR according to number of working hours instead of a cut point at 33% HRR. We obtained similar estimates to primary analysis on the relation between reported work ability and %HRR among males and females.

### 4.3. Implication of the Results and Future Recommendations

This study supports the use of single item work ability to determine the balance between work demands and resources among male blue-collar workers. The advantages of using single item work ability are mainly the short time needed to answer the question, its cost effectiveness, and that the results are relatively easy to interpret by occupational and health practitioners. However the results from this study underline that the interpretation based on the single work ability item should be used with care, especially among female blue-collar workers. This study supported the face validity of single item work ability with %HRR among male blue-collar workers from different occupational groups such as construction workers, cleaners, road maintenance workers, street cleaners/garbage disposers, manufacturing workers, truck drivers and workers in the health service sector, with a wide range of age, seniority, work tasks, and work demands. However, we could not establish these results among females. A potential explanation behind not obtaining the significant results among females could be their relatively low sample size (*n* = 53) in this study. Thus, future studies investigating the face validity of the single item work ability against objectively measured %HRR among females is warranted.

## 5. Conclusions

This study supports the face validity of the single work ability item among a working population of male blue-collar workers. Therefore, the self-reported work ability item seems to provide a useful measure of the relation between work demands and resources among male blue-collar workers. A similar correspondence between work ability and balance between work demands and resources was not found among females workers which need to be further investigated in larger populations.

## References

[1] Hannerz H., Tuchsen F., Spangenberg S., Albertsen K. (2004). Industrial differences in disability retirement rates in denmark, 1996–2000. Int. J. Occup. Med. Environ. Health.

[2] Lahelma E., Laaksonen M., Lallukka T., Martikainen P., Pietilainen O., Saastamoinen P., Gould R., Rahkonen O. (2012). Working conditions as risk factors for disability retirement: A longitudinal register linkage study. BMC Public Health.

[3] Ilmarinen J. (2009). Work ability—A comprehensive concept for occupational health research and prevention. Scand. J. Work Environ. Health.

[4] Tuomi K., Ilmarinen J., Seitsamo J., Huuhtanen P., Martikainen R., Nygard C.H., Klockars M. (1997). Summary of the finnish research project (1981–1992) to promote the health and work ability of aging workers. Scand. J. Work Environ. Health.

[5] Ilmarinen J., Tuomi K., Klockars M. (1997). Changes in the work ability of active employees over an 11-year period. Scand. J. Work Environ. Health.

[6] Van den Berg T.I., Elders L.A., de Zwart B.C., Burdorf A. (2009). The effects of work-related and individual factors on the work ability index: A systematic review. Occup. Environ. Med..

[7] Sell L. (2009). Predicting long-term sickness absence and early retirement pension from self-reported work ability. Int. Arch. Occup. Environ. Health.

[8] Bowling A. (2005). Just one question: If one question works, why ask several?. J. Epidemiol. Community Health.

[9] Nygard C.H., Eskelinen L., Suvanto S., Tuomi K., Ilmarinen J. (1991). Associations between functional capacity and work ability among elderly municipal employees. Scand. J. Work Environ. Health.

[10] Ahlstrom L., Grimby-Ekman A., Hagberg M., Dellve L. (2010). The work ability index and single-item question: Associations with sick leave, symptoms, and health—A prospective study of women on long-term sick leave. Scand. J. Work Environ. Health.

[11] Strijk J.E., Proper K.I., van Stralen M.M., Wijngaard P., van Mechelen W., van der Beek A.J. (2011). The role of work ability in the relationship between aerobic capacity and sick leave: A mediation analysis. Occup. Environ. Med..

[12] Johnson R.W., Mermin G.B., Resseger M. (2011). Job demands and work ability at older ages. J. Aging Soc. Policy.

[13] Mokkink L.B., Terwee C.B., Patrick D.L., Alonso J., Stratford P.W., Knol D.L., Bouter L.M., de Vet H.C. (2010). The cosmin checklist for assessing the methodological quality of studies on measurement properties of health status measurement instruments: An international delphi study. Qual. Life Res..

[14] Kwak L., Proper K.I., Hagstromer M., Sjostrom M. (2011). The repeatability and validity of questionnaires assessing occupational physical activity—A systematic review. Scand. J. Work Environ. Health.

[15] Adler N.E., Boyce T., Chesney M.A., Cohen S., Folkman S., Kahn R.L., Syme S.L. (1994). Socioeconomic status and health. The challenge of the gradient. Am. Psychol..

[16] Astrand P.-O., Rodahl K. (1986). Textbook of Work Physiology.

[17] Sogaard K., Fallentin N., Nielsen J. (1996). Work load during floor cleaning. The effect of cleaning methods and work technique. Eur. J. Appl. Physiol. Occup. Physiol..

[18] Makowiec-Dabrowska T., Bortkiewicz A., Radwan-Wlodarczyk Z., Koszada-Wlodarczyk W. (1992). Physiological reaction to workload in women performing manual or mental work. Pol. J. Occup. Med. Environ. Health.

[19] Tanaka H., Monahan K.D., Seals D.R. (2001). Age-predicted maximal heart rate revisited. J. Am. Coll. Cardiol..

[20] Mackinnon L.T., Ritchie C.B., Hooper S., Abernethy P., Mackinnon L., Ritchie C.B., Hooper S., Abernethy P. (2003). Exercise Management: Concepts and Professional Practice.

[21] Pollock M.L., Foster C., Rod J.L., Wible G. (1982). Comparison of methods for determining exercise training intensity for cardiac patients and healthy adults. Adv. Cardiol..

[22] Ilmarinen J. (1992). Job design for the aged with regard to the decline in their maximal aerobic capacity: Part i-guidelines for the practitioner. Int. J. Ind. Ergon..

[23] strand P.O. (1956). Human physical fitness with special reference to sex and age. Physiol. Rev..

[24] Cink R.E., Thomas T.R. (1981). Validity of the astrand-ryhming nomogram for predicting maximal oxygen intake. Br. J. Sports Med..

[25] Gram B., Holtermann A., Bultmann U., Sjogaard G., Sogaard K. (2012). Does an exercise intervention improving aerobic capacity among construction workers also improve musculoskeletal pain, work ability, productivity, perceived physical exertion, and sick leave? A randomized controlled trial. J. Occup. Environ. Med..

[26] Holtermann A., Mortensen O.S., Burr H., Sogaard K., Gyntelberg F., Suadicani P. (2010). Physical demands at work, physical fitness, and 30-year ischaemic heart disease and all-cause mortality in the copenhagen male study. Scand. J. Work Environ. Health.

[27] Holtermann A., Hansen J.V., Burr H., Sogaard K. (2010). Prognostic factors for long-term sickness absence among employees with neck-shoulder and low-back pain. Scand. J. Work Environ. Health.

[28] Neupane S., Miranda H., Virtanen P., Siukola A., Nygard C.H. (2011). Multi-site pain and work ability among an industrial population. Occup. Med..

[29] De Vries H.J., Reneman M.F., Groothoff J.W., Geertzen J.H., Brouwer S. (2013). Self-reported work ability and work performance in workers with chronic nonspecific musculoskeletal pain. J. Occup. Rehabil..

[30] Pejtersen J.H., Kristensen T.S., Borg V., Bjorner J.B. (2010). The second version of the copenhagen psychosocial questionnaire. Scand. J. Public Health.

[31] Kristiansen J., Korshoj M., Skotte J.H., Jespersen T., Sogaard K., Mortensen O.S., Holtermann A. (2011). Comparison of two systems for long-term heart rate variability monitoring in free-living conditions—A pilot study. Biomed. Eng. Online.

[32] Tudor-Locke C., Johnson W.D., Katzmarzyk P.T. (2009). Accelerometer-determined steps per day in us adults. Med. Sci. Sports Exerc..

[33] Strath S.J., Swartz A.M., Bassett D.R., O’Brien W.L., King G.A., Ainsworth B.E. (2000). Evaluation of heart rate as a method for assessing moderate intensity physical activity. Med. Sci. Sports Exerc..

[34] Karvonen J., Vuorimaa T. (1988). Heart rate and exercise intensity during sports activities. Practical application. Sports Med..

[35] Wu H.C., Wang M.J. (2002). Relationship between maximum acceptable work time and physical workload. Ergonomics.

[36] Krause N., Brand R.J., Kaplan G.A., Kauhanen J., Malla S., Tuomainen T.P., Salonen J.T. (2007). Occupational physical activity, energy expenditure and 11-year progression of carotid atherosclerosis. Scand. J. Work Environ. Health.

[37] Michael E.D., Hutton K.E., Horvath S.M. (1961). Cardiorespiratory responses during prolonged exercise. J. Appl. Physiol..

[38] Rodgers S.H., Kenworth D.A., Eggleton E.M. (1986). Ergonomic Design for People at Work.

[39] Vandenbroucke J.P., von Elm E., Altman D.G., Gotzsche P.C., Mulrow C.D., Pocock S.J., Poole C., Schlesselman J.J., Egger M. (2007). Strengthening the reporting of observational studies in epidemiology (strobe): Explanation and elaboration. PLoS Med..

[40] Rosenthal J.A. (1996). Qualitative descriptors of strength of association and effect size. J. Soc. Serv. Res..

[41] Merrill R.M. (2011). Principles of Epidemiology Workbook: Exercises and Activities.

[42] Robergs R.A., Landwehr R. (2002). The surprising history of the “hrmax = 220-age” equation. J. Exerc. Physiol. Online.

[43] Christensen C.C., Frey H.M., Foenstelien E., Aadland E., Refsum H.E. (1983). A critical evaluation of energy expenditure estimates based on individual o2 consumption/heart rate curves and average daily heart rate. Am. J. Clin. Nutr..

[44] Glaros A.G., Williams K., Lausten L. (2008). Diurnal variation in pain reports in temporomandibular disorder patients and control subjects. J. Orofac. Pain.

[45] Holtermann A., Jørgensen M., Gram B., Christensen J., Faber A., Overgaard K., Ektor-Andersen J., Mortensen O., Sjøgaard G., Søgaard K. (2010). Worksite interventions for preventing physical deterioration among employees in job-groups with high physical work demands: Background, design and conceptual model of finale. BMC Public Health.

[46] Aittomaki A., Lahelma E., Roos E. (2003). Work conditions and socioeconomic inequalities in work ability. Scand. J. Work Environ. Health.

[47] Pohjonen T., Ranta R. (2001). Effects of worksite physical exercise intervention on physical fitness, perceived health status, and work ability among home care workers: Five-year follow-up. Prev. Med..

[48] Nielsen J. (1999). Employability and workability among danish employees. Exp. Aging Res..

[49] Sell L., Faber A., Søgaard K., Masaharu K. (2008). Impacts from Occupational Risk Factors on Self Reported Reduced Work Ability among Danish Wage Earners. Promotion of Workability towards Productive Aging: Selected papers of the 3rd International Symposium on Workability, Hanoi, Vietnam, 22–24 October 2007.

[50] Fletcher G.F., Balady G., Froelicher V.F., Hartley L.H., Haskell W.L., Pollock M.L. (1995). Exercise standards: A statement for healthcare professionals from the american heart association. Circulation.

